# A Comparative Review of Graphene and MXene-Based Composites towards Gas Sensing

**DOI:** 10.3390/molecules29194558

**Published:** 2024-09-25

**Authors:** Pushpalatha Vijayakumar Vaishag, Jin-Seo Noh

**Affiliations:** Department of Physics, Gachon University, 1342 Seongnamdaero, Sujeong-gu, Seongnam-si 13120, Gyeonggi-do, Republic of Korea; vaishagpnair@gmail.com

**Keywords:** graphene, MXenes, metal oxides, composites, gas sensors

## Abstract

Graphene and MXenes have emerged as promising materials for gas sensing applications due to their unique properties and superior performance. This review focuses on the fabrication techniques, applications, and sensing mechanisms of graphene and MXene-based composites in gas sensing. Gas sensors are crucial in various fields, including healthcare, environmental monitoring, and industrial safety, for detecting and monitoring gases such as hydrogen sulfide (H_2_S), nitrogen dioxide (NO_2_), and ammonia (NH_3_). Conventional metal oxides like tin oxide (SnO_2_) and zinc oxide (ZnO) have been widely used, but graphene and MXenes offer enhanced sensitivity, selectivity, and response times. Graphene-based sensors can detect low concentrations of gases like H_2_S and NH_3_, while functionalization can improve their gas-specific selectivity. MXenes, a new class of two-dimensional materials, exhibit high electrical conductivity and tunable surface chemistry, making them suitable for selective and sensitive detection of various gases, including VOCs and humidity. Other materials, such as metal-organic frameworks (MOFs) and conducting polymers, have also shown potential in gas sensing applications, which may be doped into graphene and MXene layers to improve the sensitivity of the sensors.

## 1. Introduction

Gas sensors have become essential tools in many fields, including healthcare, environmental monitoring, and industrial safety, in today’s technologically evolved society. Essentially, a gas sensor is a device that senses the amount or presence of gases in a given space and transforms these data into an electrical signal. The safety, well-being, and health of people as well as the environment depend on this fairly straightforward procedure. The significance of gas sensors cannot be overemphasized. These sensors are essential in industrial environments because they monitor and identify dangerous gases, which helps to avert mishaps and guarantee worker safety. For example, in chemical plants, gas sensors can detect leaks of toxic or flammable gases like ammonia or methane, triggering alarms and enabling timely interventions to avoid disasters. Another significant field where gas sensors are highly influential is environmental monitoring [1]. These sensors are essential for tracking the amount of pollution in cities, identifying the sources of harmful pollutants and evaluating air quality. Gas sensors give accurate and up-to-date information on greenhouse gases and other pollutants, which helps with the enforcement of laws and the promotion of sustainable practices. Concerns about climate change and environmental degradation are becoming more and more relevant. In the field of health care, gas sensors are also necessary for respiratory monitoring and diagnostic applications [2]. They help in the detection and treatment of respiratory disorders by measuring vital factors such as patients’ oxygen and carbon dioxide levels in medical equipment [3]. Moreover, innovative gas sensing technologies are being explored for the early detection of diseases through breath analysis, showcasing the potential of these sensors in non-invasive medical diagnostics. The rapid advancements in materials science and nanotechnology have propelled the development of highly sensitive, selective fabric gas sensors, wearable gas sensors, colorimetric sensors, and optical gas sensors [4,5,6,7,8].

Common gases like hydrogen sulfide (H_2_S), nitrogen dioxide (NO_2_), and ammonia (NH_3_) are sensed using different materials and strategies. Hydrogen sulfide (H_2_S) has a distinctly “rotten egg” odor that makes it very poisonous and colorless. Haze can irritate the eyes, nose, and throat even at low concentrations. Moderate quantities (20 to 100 ppm) in acute exposure may cause more serious health consequences such as headache, nausea, dizziness, and irritation of the eyes and airways [9,10]. Elevated levels (more than 100 ppm) provide a significant hazard since they can swiftly result in unconsciousness, respiratory paralysis, and fatality. Respiratory difficulties and other health concerns might arise over time with prolonged exposure to lower levels. In sectors like natural gas, wastewater treatment, and petroleum refining, where H_2_S is extremely harmful, constant monitoring of H_2_S levels is essential. Nitrogen dioxide (NO_2_), a reddish-brown, extremely poisonous gas with an unpleasant odor, is another significant gas. It is a major air pollutant that is mostly caused by industrial operations and automobile emissions. There are major health hazards associated with NO_2_, especially to the respiratory system. Acute exposure can worsen asthma and other chronic respiratory disorders and cause coughing, chest discomfort, and irritation of the nose, eyes, and throat. Furthermore, NO_2_ is a precursor to fine particulate matter and ground-level ozone, both of which have detrimental effects on the environment and human health. Owing to its toxicity and pervasiveness, NO_2_ levels in urban and industrial environments must be regularly monitored to safeguard public health and guarantee adherence to air quality regulations [11,12].

Currently, many materials have been researched and developed for gas sensors, each offering unique advantages in terms of sensitivity, selectivity, and response time. Traditional metal oxides such as tin oxide (SnO_2_), zinc oxide (ZnO), metal oxides combined with carbon families (CNT-TiO_2_), and rare metal oxides have been widely used due to their high sensitivity and relatively simple fabrication processes [13,14,15,16,17,18,19,20]. Incorporating metal oxides into MXene- and graphene-based composites offers several advantages, particularly in gas sensing applications. They are widely known for their potent gas adsorption abilities and semiconducting characteristics, particularly when oxygen is present. When exposed to gases, these oxides readily absorb gas molecules and promote the formation of electron depletion layers (EDLs), which significantly alter conductivity. In addition to these composites, polymers may be utilized to provide other benefits, including flexibility, tunable qualities, and resistance to humidity, which increase the material’s usefulness in actual gas sensing situations. These materials work well for detecting nitrogen dioxide (NO_2_) and carbon monoxide (CO), among other gases. Cutting-edge materials with remarkable mechanical, electrical, and thermal capabilities, including graphene and its variants, have attracted a lot of interest. Graphene-based sensors can detect low levels of gases such as hydrogen sulfide (H_2_S) and ammonia (NH_3_) because of their high sensitivity and quick reaction times. Materials like graphene and MXenes are at the forefront of this evolution, offering superior performance in detecting a wide range of gases at low concentrations [21]. The ultra-thin texture of graphene makes it extremely sensitive to gas adsorption, and resulting variations in conductivity and electron density indicate the presence of gas. Its fast electron mobility provides rapid response and recovery times. The adjustable surface chemistry of MXenes, on the other hand, makes gas adsorption and desorption more effective. This is due to terminations like -OH, -O, or -F. These advancements enhance the accuracy and reliability of gas sensors and expand their applicability in various fields like biosensors, drug delivery systems, catalysis, etc. [22,23,24,25]. Furthermore, the functionalization of graphene can improve its gas-specific selectivity. Metal–organic frameworks (MOFs) are a potential new class of materials [26]. These porous structures offer high surface areas and tunable chemical environments, which can be tailored for selective gas adsorption and detection [27,28]. MOFs have shown great potential in sensing applications for gases like carbon dioxide (CO_2_) and volatile organic compounds (VOCs) [29,30]. MXenes, a relatively new class of two-dimensional materials, have also shown excellent gas sensing capabilities [31,32,33]. Their high electrical conductivity and adjustable surface chemistry make them suitable for highly selective and sensitive detection of a wide range of gases, including NH_3_ and NO_2_ [34,35]. Additionally, conducting polymers like polypyrrole and polyaniline are being researched because of their versatility and ease of integration into wearable and portable sensing devices [36,37,38]. In graphene and MXene-conducting polymer composites, the strong conductivity and vast surface area of graphene and MXenes are combined with the conducting polymer’s capacity to undergo reversible doping and de-doping during gas exposure to enhance sensitivity and selectivity. The polymers improve gas interaction even further by adding flexibility and more functional groups. These composites provide better stability, sensitivity, and selectivity for gas sensing under a variety of circumstances. The advancement of environmental awareness, health monitoring, and personal safety depends heavily on wearable gas sensors. These sensors allow for the real-time detection of hazardous gases in the user’s immediate environment by blending those seamlessly with apparel or accessories. They reduce health hazards by warning workers of unsafe quantities of gases like hydrogen sulfide or carbon monoxide in industrial environments like mining and chemical manufacturing. Furthermore, wearable gas sensors are very important in the medical field. They can track exhaled biomarkers and monitor respiratory problems, which helps doctors to treat diseases like diabetes and asthma. In addition to improving health and safety, they raise environmental consciousness by enabling people to track the state of the air, identify toxins, and take well-informed action to limit their exposure to dangerous gases in contaminated metropolitan areas. Wearable gas sensors are crucial for increasing safety and well-being in daily life because of their capacity to quietly and continually monitor these elements. These materials are appropriate for applications requiring flexible and lightweight sensors because they change their electrical characteristics in response to gas exposure [39]. Even though MOFs and CNTs are all promising candidates for gas sensing due to their unique properties, they differ in terms of sensitivity and selectivity. Due to its high electrical conductivity and huge surface area, graphene is recognized for its exceptional sensitivity, which allows it to detect even single-molecule adsorption. But until functionalized, pristine graphene is not selective. MXenes provide higher sensitivity and greater selectivity than graphene due to their metallic conductivity and modifiable surface chemistry, particularly for gases like NH_3_ and NO_2_. Their hydrophilic nature and surface terminations, which improve gas interaction, are primarily responsible for this. On the other hand, MOFs are excellent at gas selectivity because of their very porous and adjustable structure, which enables them to be designed for certain gases like CO_2_ and VOCs. Despite their high selectivity, however, MOFs frequently suffer with stability in extreme environments and shorter reaction times. CNTs are just as sensitive as graphene due to their high conductivity and surface area. Functionalization is necessary for increased selectivity. All things considered, MXenes balance sensitivity and selectivity, which makes them very good for gas sensing applications, especially in environments with fluctuating circumstances where other materials might not hold up. In the meantime, functionalization is still essential to maximizing the selectivity and sensitivity of MOFs and CNTs in real-world applications. In summary, the investigation of many materials, such as metal oxides, graphene, MOFs, MXenes, CNT, and conducting polymers, has led to substantial breakthroughs in the creation of gas sensors [40]. These materials all have different advantages that can work together to provide gas-sensing technologies that are extremely selective, adaptable, and sensitive enough for various uses.

In this work, we reviewed the fabrication technique for graphene and MXenes composites and the applications and functionalities of these materials toward gas sensing. Our objective is to add to the corpus of synthetic methodologies, gas sensor types that have been used recently, and their mechanisms. We also offer a thorough introduction to graphene- and MXene-based gas sensors.

## 2. Synthesis

### 2.1. Synthesis of Graphene

Several steps are involved in the manufacture of graphene. Conductive and semiconducting nanomaterials have been included in the prototypes in addition to graphene to improve their selectivity and specificity towards particular gases. For instance, graphene is often coupled with metal oxides like SnO_2_ and ZnO to target certain gases like CO and NO_2_. More accurate gas detection is made possible by the semiconductor properties of these metal oxides, where gas molecules adsorb on the surface and cause changes in resistance. Furthermore, when combined with graphene, materials like MoS_2_ and TiO_2_ have demonstrated enhanced sensitivity and selectivity toward gases like NH_3_ and H_2_S. These combinations of nanocomposites take advantage of the high conductivity and surface area of graphene and the special electrical characteristics of semiconductors simultaneously to improve the overall gas sensing capability. Higher selectivity for the targeted gases is the outcome of this synergy, enhancing the practical applicability of these prototypes. In this synthesis part, we focus on the different synthesis techniques of graphene and graphene-based materials that might be advantageous for gas sensors [41,42,43].

Natarajamani et al. prepared graphene oxide (GO) using the electrochemical exfoliation method from the use-and-throw battery graphite rod [44]. Here, the graphite rod acts as an anode, and the copper rod acts as a cathode. These electrodes were placed 2 cm apart and dipped in 50 mL of ammonium sulfate. An applied voltage of 10 V was used to achieve electrochemical exfoliation. In another work carried out by R. Singh et al., GO was synthesized using a modified Hummers method, where the graphite flakes were mixed with sulfuric and orthophosphoric acids, stirred in an ice-water bath, and combined with potassium permanganate below 30 °C [45]. After adding distilled water and hydrogen peroxide, the mixture was ultrasonicated, and GO precipitates were collected, washed to neutral pH, and air-dried for 24 h. GO was then reduced to reduced graphene oxide (rGO) using hydrazine hydrate in a hydrothermal process. Another versatile method for synthesizing materials based on graphene is chemical vapor deposition (CVD). In the research conducted by Gautam et al., they examined the gas sensing characteristics of graphene, which was created by the CVD technique [46]. They used methane and hydrogen gasses in the CVD process to create graphene sheets on a copper substrate. A pure copper substrate was first cleaned and heated in a tube furnace using a hydrogen–argon combination for an hour at 1060 °C to eliminate any organic impurities and oxide coatings. The next step involved flowing a combination of argon and methane over the substrate for 10–12 min to deposit graphene. The spin-coating technique was used to coat a PMMA layer over deposited graphene on copper. Then, 1 mg of aqueous FeCl_3_ solution was used to etch the copper substrate. Subsequently, the graphene layer was shifted to a SiO_2_/Si substrate, and an acetone rinse was used to eliminate the PMMA layer. UV spectroscopy was used to verify the thickness of the thermally produced SiO_2_, which was found to be 118 nm. In a similar vein, superior graphene sheets were created by cooling the system to ambient temperature and changing the gas composition to hydrogen after the deposit.

**Figure 1:**
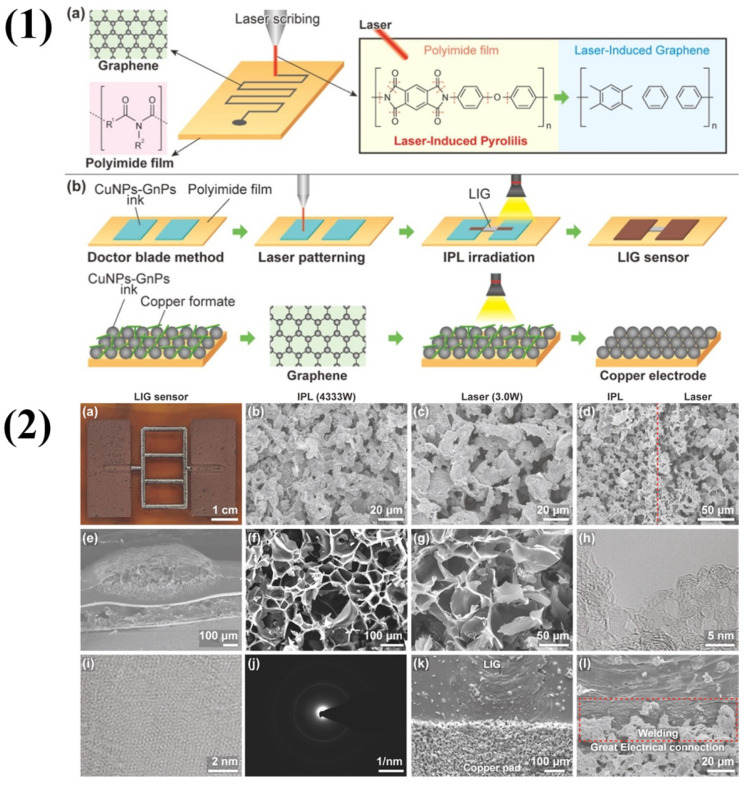
(**1**) A schematic illustrating the fabrication process of an LIG sensor. (**a**) The mechanism involved in the formation of LIG on the PI substrate. (**b**) A schematic demonstrating the realization of the LIG sensor using laser patterning followed by an IPL-sintered copper electrical pad on the PI substrate. (**2**) Morphological analysis of the LIG sensor. (**a**) Photograph of the fabricated LIG sensor. (**b**,**c**) FESEM images of IPL and laser-sintered CuNPs, respectively. (**d**) FESEM micrograph depicting the interface between IPL-sintered CuNPs and laser-sintered CuNPs. FESEM micrographs of the LIG (**e**) at lower magnification and (**f**,**g**) at higher magnification. (**h**,**i**) HR-TEM images of the LIG. (**j**) SAED pattern were obtained from the LIG. FESEM micrographs illustrating the interface between LIG and copper electrode (**k**) at lower magnification and (**l**) at higher magnification. Reproduced from Paeng, C.; Shanmugasundaram, A.; We, G.; Kim, T.; Park, J.; Lee, D.W.; Yim, C. Rapid and Flexible Humidity Sensor Based on Laser-Induced Graphene for Monitoring Human Respiration. *ACS Appl. Nano Mater.* **2024**, *7*, 4772–4783 [48].

Santos-Ceballos et al. synthesized 3D graphene networks utilizing a laser, which is known as the laser-induced graphene (LIG) method [47]. A flexible LIG electrode was fabricated using direct laser drawing on a commercially available polyimide film (50 µm thickness) attached to a PET sheet. A CO_2_ pulsed laser system (48-2, SYNRARD) with a wavelength of 10.6 µm and a maximum power of 25 W was used for LIG synthesis. The laser was focused onto the polyimide surface through a lens with a focal length of 74 mm. The laser beam scanned the substrate at a constant speed of 200 mm/s, a frequency of 12 kHz, and a power level of 12%. This method was first introduced by Prof. Tour in 2014 [28]. This is a one-step, simple, scalable, and low-cost approach for producing and patterning porous graphene films with 3D networks from commercial polymer films. This material exhibits high porosity, excellent electrical conductivity, good mechanical flexibility, high thermal stability, and outstanding electrochemical performance. Similarly, Paeng et al. explained the formation of LIG in their paper [48]. When the polyimide (PI) absorbs the laser energy, causing localized heating, photochemical processes that produce laser-induced graphene (LIG) on the PI substrate are activated. Upon absorption of the laser energy by the imide groups and aromatic moieties of PI’s structure, internal chemical bonds such as C−C and C=O break. Carbon-rich species are created because of the PI’s pyrolysis and heat-induced thermal breakdown induced by localized heating. When these carbon atoms rearrange and recombine, the surface of the PI film is coated with a structure that looks like graphene. An LIG sensor fabrication process is shown in Figure 1(1,2) shows the morphological analysis of the fabricated LIG sensor. When manufacturing graphene, LIG has several benefits over electrochemical exfoliation and Hummer’s method. LIG is a quick, easy, and eco-friendly method requiring a substrate and a laser. It does not use toxic chemicals and produces very little waste. In comparison, electrochemical exfoliation is a cleaner approach but requires potentially dangerous electrolytes; the Hummers method is labor-intensive and time-consuming and contains hazardous chemicals. LIG is also incredibly versatile and scalable, quickly adjusting to a wide range of substrates, including flexible materials. Additionally, compared to bulk graphene oxide created by the Hummers technique, which requires additional processing, LIG permits fine patterning directly on the substrate, which is advantageous for electrical and sensor applications. Another interesting graphene-based material in gas sensing is graphene quantum dots (GQDs). Malathi et al. synthesized nitrogen-doped graphene quantum dots (NGQDs) for NO_2_ gas sensing at room temperature (RT) in a very simple manner [11]. In the synthesis of NGQDs, citric acid and urea play crucial roles. The carbon source for the graphene quantum dot production is citric acid. Citric acid carbonizes during the autoclave’s heating phase, creating the quantum dots’ carbon framework and facilitating the reduction process. Its organic acid content aids in regulating the quantum dot size as well. The nitrogen source used to dope the graphene quantum dots is urea. Nitrogen doping occurs when urea breaks down and releases species that contain nitrogen. These species then integrate into the carbon lattice of the quantum dots. The graphene quantum dot’s electrical characteristics are much improved by this nitrogen addition.

**Figure 2:**
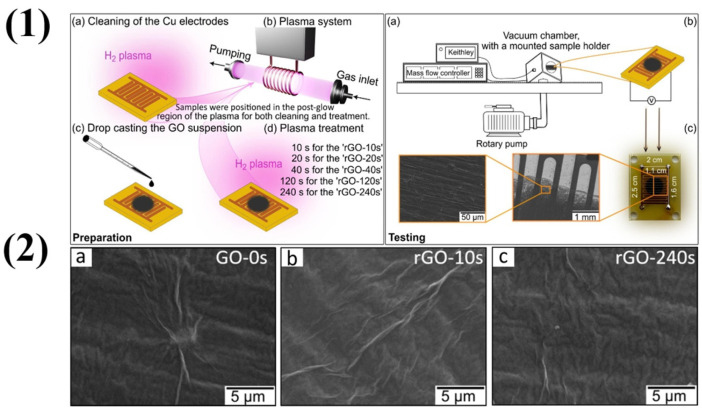
(**1**) Left panel: Schematic representation of the steps undertaken for the sensors’ preparation and treatment. Right panel: (**a**) Scheme of the system employed for NH sensing measurements; (**b**) a drawing that symbolizes the sensor’s connection to the Keithley multimeter; (**c**) digital image of the sensor with magnified sections featuring SEM images of the drop-cast GO suspension, providing visual evidence of the even distribution of GO suspension and electrode coverage. (**2**) SEM micrographs of sensor surface after plasma treatment: (**a**) 0 s (GO), (**b**) 10 s (rGO), and (**c**) 240 s (rGO), pinpointing no apparent damages on the surface. Reproduced from Kurtishaj Hamzaj, A.; Donà, E.; M Santhosh, N.; Shvalya, V.; Košiček, M.; Cvelbar, U. Plasma-Modification of Graphene Oxide for Advanced Ammonia Sensing. *Appl. Surf. Sci.* **2024**, *660*, 1–11 [50].

Balakrishnan et al. also synthesized NGQDs for nitrobenzene gas sensors, but instead of using urea, they used ethylenediamine (EDA) as the primary source of nitrogen [49]. In this work, they sensed the nitrobenzene gas fluorometrically. GQDs are especially well suited for cutting-edge applications because they have many benefits over traditional graphene nanosheets (GNs). GQDs have a larger surface area-to-volume ratio than other materials because of their small size typically less than 10 nm, which improves their reactivity and interaction with other materials and is advantageous for sensing applications. Hamzaj et al. studied a controlled plasma-assisted method for quick and environmentally friendly GO film surface reduction that was especially designed for room-temperature ammonia (NH_3_) detection at ppm levels [50]. A reduction process aided by plasma was utilized to convert GO into rGO. GO was initially used for sensor chips that had already undergone hydrogen plasma pretreatment, which improved electrode contact by removing the oxide layer from the copper surface. The chips were plasma-cleaned, and then, the GO ink was drop-cast onto them after being diluted in a 40% ethanol/distilled water solution. The chips were then allowed to dry overnight. However, these chips’ high resistance at first made them unsuitable for ammonia detection. The reduction in GO to rGO induced by this plasma treatment facilitated the minimization of resistance. The chips were treated with hydrogen plasma using a low-pressure radio frequency inductively coupled plasma system to increase conductivity. The system was operated at 100 sccm of hydrogen flow rate, 32 Pa of pressure, and 100 W of energy. To avoid overheating and surface damage, the chips were positioned in the post-glow zone of the plasma and subjected to varying treatment durations (10 s, 20 s, 40 s, 120 s, and 240 s), as illustrated in Figure 2(1). SEM micrographs unequivocally demonstrate that no plasma damage occurred to the rGO samples, as shown in Figure 2(2).

### 2.2. Synthesis of MXenes

MXenes are a family of two-dimensional materials derived from layered transition metal carbides, nitrides, or carbonitrides, known as MAX phases. The synthesis of MXene includes many aspects. The most common synthesis method involves selectively etching out the A element (typically aluminum or another elements of group 13 or 14) from the MAX phase to obtain the two-dimensional MXene structure. MXenes’ layered structure, which is made up of alternating layers of carbon or nitrogen and transition metals, is essential to how they interact with gas molecules, changing their resistance or conductivity. The surfaces of MXenes have widely varying functional groups typically comprising -O, -OH, and -F, which affect how gases interact with them. The adsorption of gas molecules onto the MXene surface might occur by physical adhesion or chemical reaction. Through modifications to the band structure or charge carrier concentration, this interaction influences the electronic characteristics of the MXenes. For example, charge transfer between the gas molecules and MXenes can occur because of gas adsorption, and this can produce measurable changes in electrical resistance or conductivity. MXenes are very responsive to changes in their surroundings because of their high conductivity and flexibility in surface modification, which increases their efficacy as gas sensors.

The first reported synthesis of Ti_3_C_2_T*_x_* MXene begins with the preparation of Ti_3_AlC_2_ precursor material (MAX phase). This is achieved by ball-milling Ti_2_AlC and TiC powders in a 1:1 molar ratio for 24 h using zirconia balls. The resulting mixture is then heated to 1350 °C for 2 h under an argon atmosphere, forming a loosely densified chunk that is subsequently crushed using a mortar and pestle. The ground powder is immersed in about 100 mL of 50% concentrated hydrofluoric acid (HF) solution at room temperature for 2 h. This etching process removes the aluminum layers from the Ti_3_AlC_2_, resulting in the formation of Ti_3_C_2_T*_x_* MXene. The suspension is washed multiple times with deionized water and centrifuged to separate the MXene powders. In some cases, to align the flakes and produce free-standing discs, the treated MXene powders are cold-pressed at a load corresponding to a stress of 1 GPa in a steel die. This process yields high-quality MXene with desirable properties for various applications. The possible reactions for the formation of Ti_3_C_2_T*_x_* MXene and the surface functionality are as follows [51]:

Ti_3_AlC_2_ + 3HF = AlF_3_ + 3/2 H_2_ + Ti_3_C_2_ (1)

Ti_3_C_2_ + 2H_2_O = Ti_3_C_2_(OH)_2_ + H_2_ (2)

Ti_3_C_2_ + 2HF = Ti_3_C_2_F_2_ + H_2_ (3)

Ta et al. synthesized Ti_3_C_2_T*_x_* MXene by selectively etching aluminum from Ti_3_AlC_2_ using hydrofluoric acid (HF) [52]. They initially took 1 g of Ti_3_AlC_2_ and gradually added 32 mL of HF in a cool water bath to manage the exothermic reaction. This mixture was then heated in an oil bath at 50 °C and stirred at 500 rpm for 24 h to fully etch the aluminum layer. After the reaction, the solution was filtered and centrifuged at 8000 rpm for 10 min to remove unreacted material. The residue was repeatedly washed with deionized water using a vacuum pump until the pH reached approximately 6–7. Finally, the product was freeze-dried overnight, yielding pristine Ti_3_C_2_T*_x_* MXene powder. A combination of lithium fluoride (LiF) and hydrochloric acid (HCl) was also utilized as an agent to produce in situ HF. For example, Liu et al. employed this in situ HF technique for materializing NO_2_ gas sensors based on SnO_2_/Ti_3_C_2_T*_x_* composite [53]. The LiF-HCl mixture was combined with Ti_3_AlC_2_ and agitated for 48 h at 38 °C. The resultant solution was rinsed using deionized water and 1 M hydrochloric acid until the pH reached 6. To obtain 2D Ti_3_C_2_T*_x_* sheets, the resulting dark green supernatant was collected and freeze-dried.

Akhtar et al. used 38% hydrochloric acid and ammonium fluoride (NH_4_F) to synthesize Ti_3_C_2_T*_x_* [54]. They slowly added 0.5 g of Ti_3_AlC_2_ to the mixture solution and kept stirring. After three days, the mixture was washed multiple times with ethanol and water using a centrifuge until the supernatant reached a pH of approximately 7. The final product was dried for 5 h in an oven at 80 °C. It is easier to synthesize MXene using HF. It is a straightforward method for selective etching, but it has several drawbacks, including toxicity, environmental risk, and an irregular etching process that can result in inconsistencies in the geometry and quality of MXene. Compared to MXene synthesized using HF, MXene synthesized with a combination of LiF/NH_4_F and HCl had larger lateral dimensions and fewer nano-scale defects. The addition of Li^+^ ions and H_2_O molecules resulted in a reduction in the interaction strength between MXene layers and an increase in the interlayer spacing. This method facilitates the easier separation of MXene layers and improves the overall quality and performance of the MXene [34].

**Figure 3:**
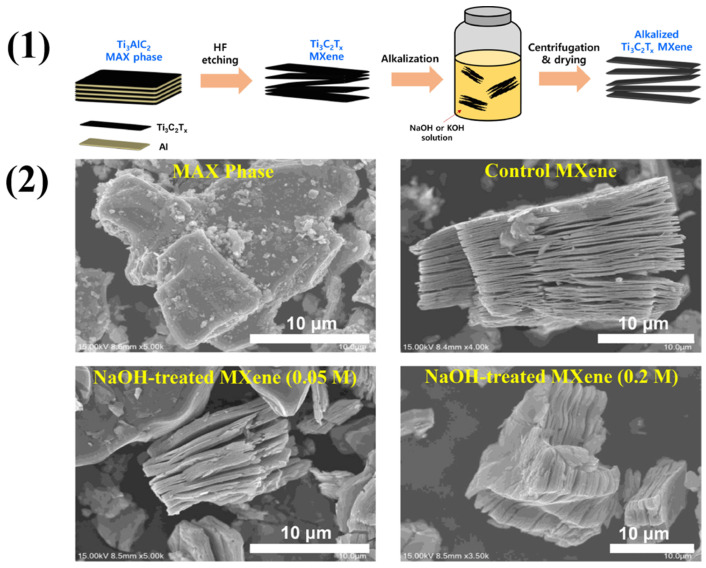
(**1**) Schematic procedures for alkalization of Ti_3_C_2_T*_x_* MXene. (**2**) SEM images of Ti_3_AlC_2_ MAX phase, control Ti_3_C_2_T*_x_* MXene, 0.05 M NaOH-treated MXene, and 0.2 M NaOH-treated MXene. Reproduced from Bae, Y.H.; Park, S.; Noh, J.S. Control of Electrical Properties of Ti_3_C_2_T*_x_* Mediated by Facile Alkalization. *Surfaces and Interfaces* **2023**, *41*, 103,258 [55].

**Figure 4:**
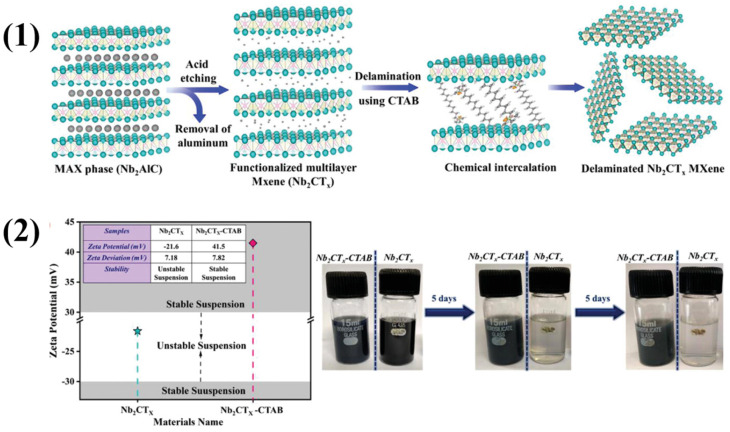
Diagrammatic representation of (**1**) material preparation and (**2**) zeta potential results of Nb_2_CT*_x_* and Nb_2_CT*_x_*-CTAB together with stability test results of prepared samples. Reproduced from Rathi, K.; Arkoti, N.K.; Pal, K. Fabrication of Delaminated 2D Metal Carbide MXenes (Nb_2_CT*_x_*) by CTAB-Based NO_2_ Gas Sensor with Enhanced Stability. *Adv. Mater. Interfaces* **2022**, *9*, 1–10 [56].

Major factors that affect the activities of MXenes include surface functionalities, interlayer spacing, conductivity, and defects. Variations in these factors also affect the gas-sensing properties. For example, a study conducted by Bae et al. showed how variations in surface functional groups affect the electrical characteristics of Ti_3_C_2_T*_x_* MXene through simple alkalization [55]. A scheme of MXene alkalization is illustrated in Figure 3(1), and SEM images of MAX, MXene, and alkalized MXene are shown in Figure 3(2). The interlayer gap increased, and XRD peaks were shifted to lower angles and became stronger after alkalization. The molecular characterizations showed that the surface terminations changed to -OH and =O groups over -F groups. Hall tests revealed a notable increase in sheet resistance and a rapid decrease in carrier concentration when Ti_3_C_2_T*_x_* MXenes were treated with different quantities of alkali salts, even at low alkali levels. Electronic transport properties were shown to be negatively affected by the transition from -F-rich to -OH/-O-rich terminations. The material’s capacity to detect gases is related to the electrical characteristics such as variations in conductivity and resistance. Rathi et al. synthesized Nb_2_CT*_x_* by etching away the Al layer with HF from the Nb_2_AlC MAX phase (48 h at 55 °C) [56]. The resultant solution was centrifuged and then rinsed until its pH reached 7. After that, they employed CTAB as an intercalating agent to increase the interlayer spacing of Nb_2_CT*_x_* MXene. Nb_2_CT*_x_* and CTAB were mixed in 40 mL of deionized water, sonicated for 1 h at a temperature below 20 °C in an argon atmosphere and then centrifuged for 30 min at 5000 rpm. After the HF treatment, Nb_2_CT*_x_* possessed surface functional groups like -O, -OH, and -F. Using CTAB for delamination and replacing H^+^ ions with CTA^+^ ions widened the interlayer gap on Nb-OH, as seen in Figure 4. The results show that the use of CTAB surfactant to delaminate Nb_2_CT*_x_* is an effective solution for enhancing NO_2_ gas sensing performance in terms of response, recovery, and stability and overcomes the issues of poor stability, low selectivity, and slow response and recovery compared to pristine Nb_2_CT*_x_* MXenes.

When compared to Ti_3_C_2_T*_x_* MXene, Nb_2_CT*_x_* exhibits higher resistance in RT, making it a less effective gas sensing material. As a potential solution of this issue, TMAOH was utilized by Wang et al. to delaminate multilayered MXene into few-layered MXene [57]. First, they dissolved NaF in hydrochloric acid and vigorously agitated it to create in situ HF. After that, Nb_2_AlC was gradually added to the mixture, and it was agitated for 96 h at 60 °C to convert it into Nb_2_CT*_x_* MXene by etching Al layers. Then, the mixture underwent centrifugation for 10 min at 7000 rpm, and washings were taken with deionized water to bring the pH to 6–7. The mixture was combined with 30 mL of TMAOH (25 wt%) and sonicated for 3 h in an ice bath. Later, it was cleaned with ethanol and deionized water to make a neutral pH. A few layered Nb_2_CT*_x_* MXene nanosheets were generated by lyophilizing the supernatant.

The synthesis methods of MXene and graphene are distinct, reflecting their unique properties. Typically, HF is used to selectively etch the A atom to synthesize MXenes. Other approaches use less hazardous compounds, including LiF with HCl or NH_4_F. After that, the etched MXenes are cleaned and occasionally intercalated with substances like TMAOH or CTAB to change their interlayer spacing and improve their characteristics. Although MXenes provide excellent conductivity and adjustable surface chemistry, managing toxic substances and attaining consistent layer thickness are still challenging. On the other hand, the synthesis of graphene is achieved using techniques such as chemical exfoliation (where GO is converted to rGO), mechanical exfoliation, and CVD (high-temperature decomposition of carbon-containing gases on a substrate). High-grade, flawless sheets with exceptional electrical and thermal conductivity are the goal of graphene production. It can be difficult to produce graphene of this quality and to scale up processes like mechanical exfoliation and CVD. Graphene is known for its remarkable mechanical and electrical capabilities, but MXene production techniques make them adaptable for a variety of applications by allowing for customizable surface properties and intercalation. Table 1 shows the advantages and disadvantages of different synthetic methods for developing graphene and MXene-based gas sensors.

## 3. Gas Sensors

In recent years, the development of advanced materials has revolutionized the field of gas sensing, leading to the emergence of highly sensitive and selective sensors. Among these materials, graphene and MXenes have garnered significant attention due to their exceptional properties and versatility. Graphene is a single layer of carbon atoms arranged in a two-dimensional hexagonal lattice and is renowned for its extraordinary electrical conductivity, high surface area, and mechanical flexibility. The interaction between gas molecules and the graphene surface leads to changes in electrical resistance, which can be accurately measured and used to identify and quantify various gases. Meanwhile, MXenes offer another promising approach to gas sensing. With their layered structure and high electrical conductivity, MXenes can be easily modified to enhance their chemical reactivity and selectivity towards specific gases. The ability to functionalize MXenes allows for the development of sensors with tailored responses, making them highly effective for detecting trace amounts of gases in various environments. Because of their remarkable qualities, both graphene and MXene-based gas sensors provide several benefits in industries. However, it is also true that they have some difference in gas sensing performance. Graphene sensors are especially useful for monitoring dangerous gases including CH_4_, CO, and VOCs in real time, owing to their great sensitivity, rapid response times, and flexibility. Their ease of functionalization makes it possible to customize them to industrial processes, improving safety and guaranteeing prompt leak detection. On the other hand, MXene sensors offer superior sensitivity and versatility for detecting low-concentration gases because to their wide surface area and strong electrical conductivity. Their ability to withstand extreme temperatures and corrosive situations makes them perfect for environmental compliance as well as process management. Collectively, these cutting-edge sensors assist safe and effective industrial operations by enhancing safety, optimizing processes, and ensuring consistent compliance with environmental laws. To improve the selectivity and specificity of the material towards a particular gas, researchers doped conducting or semiconducting nanomaterials to the graphene as well as MXenes. The interaction between the gaseous molecules and the sensors significantly alters the chemical response of the developed gas sensors. The work carried out by researchers on detecting various gases using graphene-based and MXene-based materials is highlighted in the following sections.

### 3.1. CO_2_ Gas Sensors

Carbon dioxide (CO_2_) is a major greenhouse gas contributing to global warming and climate change. For this reason, developing sensitive CO_2_ sensors is crucial for environmental and industrial applications. CO_2_ in outdoor air is safe because its concentration is relatively low (350–1000 ppm), even though it should be tightly controlled to relieve the global warming issue. Interior CO_2_ levels should be kept within this range for optimal air quality. On the other hand, higher amounts (1000–5000 ppm) may result in pain, migraines, lightheadedness, and dyspnea. Excessive concentrations (over 5000 ppm) may cause more serious health consequences, such as asphyxia death or unconsciousness, as well as disorientation and an elevated heart rate. To achieve precise, dependable, and efficient performance, it is necessary to consider many critical criteria while developing an effective CO_2_ gas sensor. To detect low CO_2_ concentrations, which are often in the parts per million (ppm) range, the sensor must have high selectivity to prevent interference from other gases such as CO, CH_4_, and NO_2_. It should also resist humidity changes.

For CO_2_ gas sensors, Casanova-Chafer et al. developed perovskite nanocrystals that were improved by oxygen plasma treatment and coupled with graphene [3]. Comparing the sensors that were treated with oxygen plasma for 5 min to those that were not, the former showed a 3-fold increase in sensing performance. Plasma treatment effectively reduces excess organic ligands on nanocrystals (NCs) by removing long-chain molecules that can isolate NC surfaces. This leads to a cleaner NC structure with increased active surface area, enhancing interaction with gases and promoting carrier transport at the graphene interface. The observed blue shift and changes in photoluminescence (PL) signal after 5 min of oxygen plasma treatment indicate the formation of additional defects, which act as active centers and improve CO_2_ reactivity. Density functional theory calculations support that these defects enhance reactivity on both graphene and CsPbBr3 perovskites. Additionally, plasma treatment introduces oxygen-containing species to the graphene surface, further improving the interaction between graphene and perovskite NCs. The treated samples showed a limit of quantification of 22.9 ppm and an LOD of 6.9 ppm. Furthermore, a study was conducted on the impact of ambient moisture on CO_2_ sensing capability, revealing a significant performance improvement caused by oxygen plasma treatment. Flexible and wearable gas-sensing devices are important for monitoring air quality and human health in daily life. Wu et al. came up with a study that introduces flexible Ti_3_C_2_T*_x_*/PANI-PP composite gas sensors [36]. The sensors were fabricated by spray-coating delaminated Ti_3_C_2_T*_x_* MXene and in situ polymerizing aniline on disposable mask substrates. These hybrid sensors exhibited a wide detection range (25–1500 ppm), reliable reproducibility, long-term stability, and excellent flexibility and selectivity. They outperformed pristine Ti_3_C_2_T*_x_* and PANI by 6.5 and 2.4 times, respectively, demonstrating a large response of 15.2% to 500 ppm CO_2_ gas. Good conductivity, greater specific surface area, and the synergistic effects of the heterojunctions between Ti_3_C_2_T*_x_* and PANI are contributing factors to the improved performance. The performance of the sensor was further investigated according to various temperatures, humidity, bending angles, and folding times. The Ti_3_C_2_T*_x_*/PANI-PP composite sensor’s potential for respiratory illness diagnosis was further demonstrated with the development of a wearable wireless Bluetooth sensing device for monitoring human breath at ambient temperature.

### 3.2. H_2_S Gas Sensors

Hydrogen sulfide (H_2_S) gas sensors are essential for ensuring safety in various environments due to the toxic and flammable nature of H_2_S. H_2_S exposure puts worker’s health in danger in industrial environments including sewage treatment facilities, mining activities, and oil and gas refineries. Even at low concentrations, prolonged exposure can cause major health problems such as headaches, dizziness, and respiratory disorders. In extreme situations, it can even be lethal. Therefore, to safeguard employees from these health risks and to maintain a safe working environment, it is essential to continuously monitor H_2_S levels using trustworthy sensors.

Bibi et al. prepared graphene aerogels (GAs) using hydrothermal reduction (GA-HR) and chemical reduction (GA-CR) to compare their properties in gas sensing applications [10]. At room temperature, they tested GA thin sheets to detect H_2_S. According to the results, GA-HR and GA-CR had H_2_S gas sensitivities of 0.255 and 0.398 ppm, respectively. Compared to GA-HR, GA-CR exhibited a slightly lower reduction degree and a more compact surface shape. Its strong sp2 character, shown by a lower ID/IG ratio in the Raman spectrum, contributed to its high electrical conductivity, leading to its high sensitivity to H_2_S. To improve gas sensing capability for H_2_S detection, Tang et al. constructed nanocomposites from porous MXene nanosheets and SnO_2_ nanoparticles in their work [9]. A three-dimensional structure was created from MXene using a sacrificial sulfur template approach to prevent flakes from restacking and decrease the operating temperature. The sensor showed a low detection limit of 100 ppb at 100 °C, a rapid response time of ~16.6 s to 50 ppm, a high sensitivity of ~3.12 ppm^−1^, and a high response of around 30.6 for 10 ppm of H_2_S. The heterojunctions that develop at the interfaces between the porous MXene nanosheets and SnO_2_ nanoparticles are responsible for the increased H_2_S sensing capability. These junctions increase the resistance variation by amplifying the surface depletion region and raising the potential barrier. Similarly, Zhang et al. developed a SnO_2_-inserted 2D-layered Ti_3_C_2_T*_x_* MXene composite through solvothermal and annealing processes [69]. This developed sensor showed rapid response and recovery times in low operating temperatures. Remarkably, the SnO_2_/Ti_3_C_2_T*_x_* composite exhibited an ultra-low theoretical limit-of-detection of 5.76 ppb and a very high sensitivity to H_2_S gas. The sensor’s optimal response to 30 ppm H_2_S reached 150, outperforming pure SnO_2_ by a factor of 11. This exceptional performance is due to the p-n heterojunction formation between Ti_3_C_2_T*_x_* MXene and SnO_2_, along with the increased active sites for gas absorption provided by the layered structure of Ti_3_C_2_T*_x_* MXene. Akhtar et al. worked with Ti_3_C_2_/Zn_2_SnO_4_-based sensor for H_2_S gas sensing, which exhibited a good response of 110 to 8 ppm, along with excellent selectivity, a minimal detection limit (LOD) of 0.01 ppm, long-term stability, and good reproducibility at room temperature [54]. The superior performance of the Ti_3_C_2_/Zn_2_SnO_4_ sensor was attributed to the heterojunction formation, higher BET surface area, and increased oxygen species.

### 3.3. NO_2_ Gas Sensors

Nitrogen dioxide (NO_2_) gas sensors are essential for ensuring health and safety, environmental monitoring, and regulatory compliance. NO_2_ is a toxic gas that can cause serious respiratory issues, making it crucial to monitor its levels in workplaces and urban areas to protect human health. Accurate sensors help assess air quality, identify pollution sources, and develop strategies to reduce pollution. Additionally, monitoring NO_2_ in cities helps manage traffic and implement pollution-reducing measures.

Govind et al. developed a high-sensitivity CuO/rGO heterostructure sensor for NO_2_ gas detection at room temperature and demonstrated remarkable performance [12]. The CuO/rGO sensor exhibited an exceptionally high sensitivity of 1004% to 5 ppm NO_2_ with a rapid response time of 9 s. This is 5.17 times better than the pristine CuO sensor. In addition, the sensor exhibited excellent linearity, remarkable repeatability, great selectivity, and long-term stability for more than 30 days. The combined effect of CuO and rGO is responsible for the improved performance. The rGO layer on top of the CuO nanosheets offers more adsorption sites and quickens the redox reaction. Its superior carrier transfer qualities further improve the responsiveness of the sensor. Additionally, chemisorbed oxygen species are present, which enhances sensing performance even more. Pan et al. undertook research to enhance NO_2_ gas sensor performance by synthesizing rGO-doped nano-octahedral α-Fe_2_O_3_ nanomaterials on indium–tin oxide (ITO) conductive glass [29]. The study developed self-supporting NO_2_ gas sensors by using solvothermal and calcination processes with MIL-88 as sacrificial templates. A particular kind of metal–organic framework (MOF) known as MIL-88 is made up of organic linkers and metal ions, usually iron. The MOF is well known for having a porous structure that permits a large surface area and adjustable characteristics, which makes it appropriate for several uses, such as sensing, gas storage, and catalysis. Its hollow and hierarchical geometry reduces detection limit and increases sensitivity, leading to the improved sensor performance. Compared to pure α-Fe_2_O_3_, the rGO/α-Fe_2_O_3_ sensor exhibited an over 8-fold improvement in response and LOD of 101 ppb. The enhanced gas response is attributed to the formation of a p-n heterostructure between rGO and α-Fe_2_O_3_ and the additional active sites for gas adsorption provided by the rGO.

Textile-based gas sensors are a kind of most interesting sensors due to their practical applicability. Jung et al. fabricated electronic textile-based gas sensors for NO_2_ detection that were developed using rGO coated on commercial cotton fabric [70]. The cotton was dipped in a GO solution and then thermally reduced at varying temperatures (190, 200, 300, and 400 °C) to assess the impact of reduction on sensing performance. The rGO-coated cotton fabric treated at 190 °C showed the highest sensing response of 45.90% to 10 ppm of NO_2_ gas at room temperature, which is attributed to its higher oxygen functional group content and the tubular structure of the cotton fabric. Similarly, Doan et al. developed highly strain-endurable gas sensors, which were integrated into the fabric from a real T-shirt using a sequential coating method [6]. The sensors incorporated multidimensional nanostructures such as rGO, ZnO nanorods, palladium nanoparticles, and silver nanowires, enabling the detection of both oxidizing and reducing gases at room temperature with varying response signs and magnitudes. Remarkably, these fabric gas sensors maintained their functionality under significant strains up to 100%, representing the highest strain tolerance in the gas sensor field. They also withstood harsh bending and twisting stresses. The sequential coating method proved to be an effective and straightforward technique for controlling the sensor size on the fabric.

**Figure 5:**
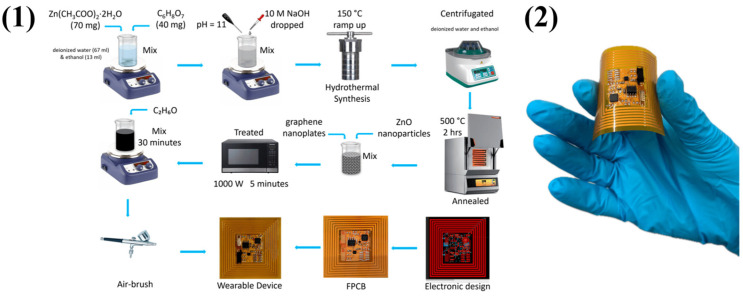
(**1**) Schematic processes for fabricating graphene/ZnO composite sensor on a flexible printed circuit board. (**2**) Picture of a wearable NFC tag system. Reproduced from Santos-Betancourt, A.; Santos-Ceballos, J.C.; Alouani, M.A.; Malik, S.B.; Romero, A.; Ramírez, J.L.; Vilanova, X.; Llobet, E. ZnO Decorated Graphene-Based NFC Tag for Personal NO_2_ Exposure Monitoring during a Workday. *Sensors* **2024**, *24*, 1431 [71].

Santos-Betancourt et al. developed a wearable gas measuring system that integrates a sensing layer with interdigitated electrodes and an electronic circuit on a flexible printed circuit board [71], as shown in Figure 5. For on-site monitoring, its innovative design offers great performance, low power consumption, and lightweight functionality. The device tracks an individual’s exposure to NO_2_ concentrations over the day by combining a chemo-resistive gas sensor with a near-field communication (NFC) tag. On a Kapton substrate, three different sensor types (bare graphene and graphene with 5 wt% zinc oxide nanoflowers and nanopillars) were tested with gold electrodes. The sensitivity and selectivity of the sensor were assessed in a variety of scenarios against NO_2_ and other interfering species. The NFC tag system is simple to attach to work vests for practical application, accumulating data in non-volatile memory every minute and operating at an average power consumption of 24.9 µW.

**Figure 6:**
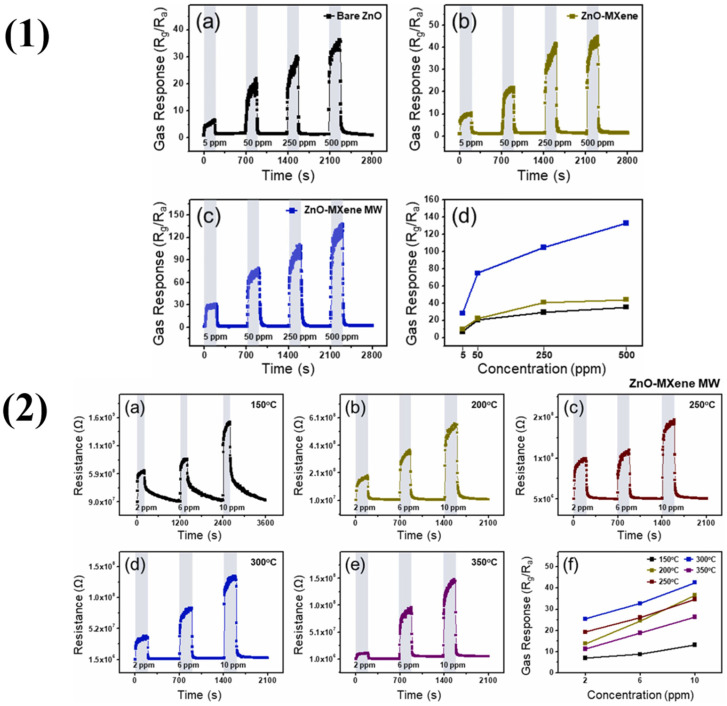
(**1**) Transient response curves of (**a**) pristine ZnO, (**b**) unirradiated ZnO/(2 wt%) Ti_3_C_2_T*_x_*, and (**c**) MW-irradiated (5 min) ZnO/(2 wt%) Ti_3_C_2_T*_x_* nanocomposite sensors to various concentration NO_2_ gas at 300 °C. (**d**) Corresponding calibration curves. (**2**) Dynamic resistance responses of MW-irradiated (5 min) ZnO/(2 wt.%) Ti_3_C_2_T*_x_* nanocomposite gas sensor to NO_2_ gas at various temperatures: (**a**) 150 °C, (**b**)200 °C, (**c**) 250 °C, (**d**) 300 °C, and (**e**) 350 °C. (**f**) Corresponding calibration curves at these temperatures. Reproduced from Shin, K.Y.; Mirzaei, A.; Oum, W.; Kim, E.B.; Kim, H.M.; Moon, S.; Kim, S.S.; Kim, H.W. Enhanced NO_2_ Gas Response of ZnO/Ti_3_C_2_T*_x_* MXene Nanocomposites by Microwave Irradiation. *Sensors Actuators B Chem*. **2024**, *409*, 135605 [73].

Peng et al. reported the preparation of MOFs-derived CuO nanoparticles on Ti_3_C_2_T*_x_* MXene nanosheets using a hydrothermal method combined with a stirring aging method, employing MOFs as sacrificial templates [72]. The binary heterostructured CuO NPs/Ti_3_C_2_T*_x_* MXene composite has many of the beneficial MOF properties, including a high porosity, a large specific surface area, an abundant number of active sites, and efficient oxidative chemical adsorption. These characteristics help explain the composite sensor’s remarkable NO_2_ gas detecting performance, which includes a reaction time of 2.84 s and a response of 38.54 to 100 ppm NO_2_ at ambient temperature. With a stability period of up to 10 weeks and an exceptionally low LOD of 30 ppb, the sensor is an extremely reliable and sensitive choice for NO_2_ gas detection in room-temperature applications. The ZnO nanoparticle’s intriguing properties make it the most widely used metal oxide in gas sensors. Shin et al. worked using ZnO/Ti_3_C_2_T*_x_* MXene nanocomposites with different MXene contents (0.5, 1, 2, and 5 wt%) to detect NO_2_ [73]. The effect of microwave (MW) irradiation time (1–8 min) on the sensor’s response was investigated. The sensor with 2 wt% Ti_3_C_2_T*_x_* MXene irradiated for 5 min exhibited the highest response of 42.65 to 10 ppm NO_2_ at 300 °C, which is shown in Figure 6. This optimal sensor also demonstrated long-term stability (over six months) and reproducibility. The enhanced NO_2_ response was attributed to the formation of ZnO/MXene Schottky barriers, increased oxygen vacancies from MW irradiation, the large surface area of the nanocomposite, and the presence of surface functional groups on MXene. This study highlighted MW irradiation as a cost-effective and accessible technique to improve gas sensing performance.

Another trend in enhancing gas sensing performance is to dope MXenes with quantum dots. Research on quantum dots (QDs)-sensitized few-layer Ti_3_C_2_T*_x_* MXene for NO_2_ detection was conducted by Feng et al. [35]. Compared to pure MXene (1.43%), this sensor demonstrated an astounding 42.05-fold increase in response (60.38% to 500 ppb NO_2_). Additionally, it showed remarkable selectivity, sustained stability, and a low LOD of 0.83 ppb, underscoring its potential for useful applications and trace-level monitoring. According to this process, QDs greatly activate the adsorption behavior and make it easier for NO_2_ molecules to transfer electrons, which improves sensing performance. Hu et al. suggested a sensitization technique that improves the performance of MXenes-based gas sensors by making use of the plasmonic photothermal effect [74]. Ti_3_C_2_T*_x_*/TiO_2_/Au heterostructures were created to detect NO_2_, and they showed a significant increase in sensitivity when activated by light of 530 nm wavelength. When exposed to 1 ppm NO_2_, the plasmonic photothermal-driven Ti_3_C_2_T*_x_*/TiO_2_/Au sensor responded 7.6 times better than Ti_3_C_2_T*_x_*/TiO_2_ and 2.34 times better than Ti_3_C_2_T*_x_*/TiO_2_/Au in the absence of light. In addition, the sensor demonstrated great selectivity, high sensitivity, and a quick response time of 5.4 s with a recovery time of 15.3 s. Increased photon usage via localized surface plasmon resonance (LSPR) and an enhanced gas-sensitive reaction pathway from the localized photothermal effect are credited with the enhancement.

### 3.4. NH_3_ Gas Sensors

Ammonia (NH_3_) gas sensors are essential due to their broad application in various industries and their critical role in ensuring safety, health, and environmental protection. Ammonia finds extensive application in industrial operations including chemical synthesis, fertilizer manufacture, and refrigeration. When present in excessive amounts, it can be harmful to human health, resulting in eye irritation, respiratory problems, and in more extreme situations, chemical burns, or respiratory failure. Thus, it is essential to monitor ammonia levels in real time to identify leaks quickly and eliminate workplace dangers.

Using the drop-casting process, Natarajamani et al. created nanostructured GO/ZnO thin films for NH_3_ gas detection at ambient temperature [44]. For 5 ppm NH_3_ at ambient temperature, the GO/ZnO thin films showed an enormous increase in sensitivity, attaining an LOD of 0.1 ppm and a response 50 times better than that of bare ZnO. The improvement in gas detecting capabilities is a result of the synergistic impact of GO and ZnO, as seen by the increased active sites and surface area resulting from GO decorating. Yogi et al. aimed to develop a simple, reliable, and effective NH_3_ sensor using rGO and ZnO nanocomposite structure, prepared via the refluxing method [75]. By employing metal-assisted chemical etching (MACE) for fabricating vertically aligned silicon nanowires (SiNWs), the rGO/ZnO nanostructure was deposited on the SiNWs structure to create a stable and selective sensor that can detect NH_3_ concentrations ranging from 0.01 ppm to 5 ppm at ambient temperature. The sensor showed responses of 21% to 0.01 ppm and 176% to 5 ppm and fast response/recovery times of 3 s/6 s and 5 s/12 s for individual concentrations. This sensor response is 17.6 times greater than pristine graphene and 44 times higher than ZnO nanoparticles. The accurate interaction at the rGO/ZnO@SiNWs Schottky heterostructure interface is responsible for the quick reaction at low LOD.

Singh et al. investigated the integration of hybrid-phase molybdenum disulfide (1T-2H MoS_2_)/nitrogen-doped graphene (NGN) with poly(3,4-ethylenedioxythiophene) (PEDOT) to improve NH_3_ gas sensing capabilities [76]. Solvothermal processing and in situ polymerization were used for preparing the PEDOT/1T-2H MoS_2_/NGN nanocomposites. The sensor PEDOT/1T-2H MoS_2_/NGN showed an estimated sensing response of 690, a response time of 34 s, a recovery time of 37 s, and an LOD of 42 ppb for NH_3_. Additionally, it had a linear response in a concentration range of 10–100 ppm. Through in situ oxidation of Ti_3_C_2_T*_x_* nanosheets on the GO surface, Shi et al. synthesized TiO_2_ nanoparticles with evenly distributed spindle-shaped nanoparticles that offer more active sites for NH_3_ adsorption [77]. Because of the distinct GO/TiO_2_ heterojunction structure, the NH_3_ sensor based on these nanocomposites responded to 100 ppm NH_3_ at ambient temperature in a manner that was almost double that of pure GO. Additionally, the sensors displayed excellent selectivity, perfect repeatability, acceptable stability, and response/recovery times of 13/33 s. Li et al. addressed and resolved the low response issue of Ti_3_C_2_T*_x_* MXene-based NH_3_ sensors by developing a gas sensor incorporating Fe_2_O_3_ nanoplates and TiO_2_ nanoparticles supported by 2D Ti_3_C_2_T*_x_* MXene [78]. An optimal sensor with 3 mL FeCl_3_ demonstrated significantly improved performance, showing a response value 3.23 times higher and shorter response/recovery times (62 s/74 s) compared to the pure Ti_3_C_2_T*_x_* MXene sensor when exposed to 100 ppm NH_3_ at room temperature. Additionally, this sensor displayed a good linear response (R^2^ = 0.95) within the range of 1–100 ppm concentration of NH_3_. The enhanced sensing capabilities are attributed to the Fe_2_O_3_/TiO_2_/Ti_3_C_2_T*_x_* MXene heterogeneous structure and unique microstructure.

Zhu et al. performed an RF magnetron sputtering for fabricating SnO_2_/Ti_3_C_2_T*_x_* heterojunction nanocomposites for NH_3_ gas sensing [79]. The heterojunctions between SnO_2_ and Ti_3_C_2_T*_x_* as well as the lamellar structure and surface porosity of the composite are beneficial to the sensor. It showed a noteworthy 13.35% response to 50 ppm NH_3_, which is 3.5 times larger than a sensor that was only based on Ti_3_C_2_T*_x_*. Furthermore, the response and recovery times were lowered to 24.9 and 86.5 s, respectively. Outstanding selectivity, repeatability, and long-term stability were also demonstrated by the SnO_2_/Ti_3_C_2_T*_x_* sensor. Wang et al. fabricated polyaniline (PANI) nanofibers supported by niobium carbide MXene (Nb_2_CT*_x_*) nanosheets, driven by a triboelectric nanogenerator (TENG) [80]. Utilizing the triboelectric effect and electrostatic induction, a TENG transforms mechanical energy into electrical energy. The phenomenon known as the triboelectric effect transpires when a specific material acquires an electrical charge after its interaction with another material and subsequent charge separation. By optimizing the spray volumes of Nb_2_CT*_x_* nanosheets, the sensor aimed to improve NH_3_ detection. A Nb_2_CT*_x_*/PANI-2 sensor, which has a spray volume of 0.1 mL, exhibited a significantly improved NH_3_-sensing speed and response as well as a good linear response across a 1–100 ppm NH_3_ range at room temperature (~25 °C) under 87.1% relative humidity (RH). The sensor’s sensitivity to humidity was reduced because the active sites are occupied by intermolecular hydrogen bonds between PANI and Nb_2_CT*_x_*. The p-n junction effect is primarily responsible for the enhanced gas sensing properties.

**Figure 7:**
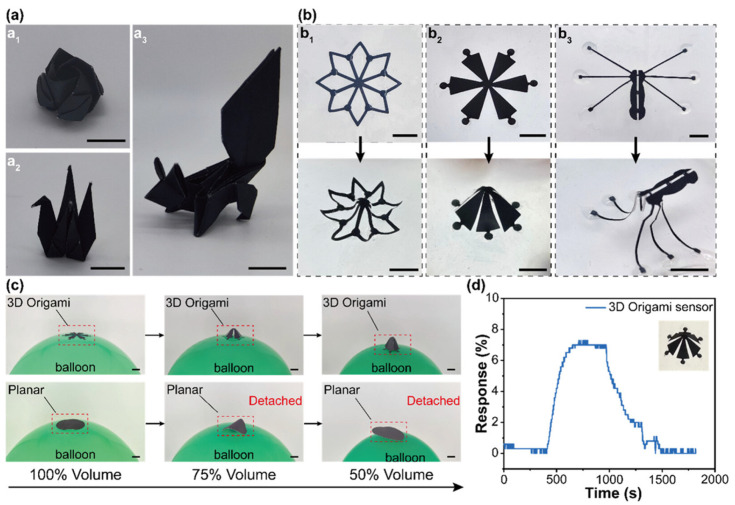
(**a**) Optical image showing three 3D MXene/gelatin origami animal models, including rose (**a1**), crane (**a2**), and squirrel (**a3**), respectively. Scale bar: 10 mm. (**b**) Optical image showing three types of 3D MXene/gelatin origamis prepared by mechanically guided compressive buckling, including an openwork octagonal structure (**b1**), a hexagonal umbrella (**b2**), and a six-legged ant (**b3**), respectively. Scale bar: 10 mm. (**c**) Detachment test of the 3D origami and the planar pattern on the surface of the shrinking balloons. Scale bar: 10 mm. (**d**) Sensing response of the proposed 3D MXene/gelatin origami sensor to 50 ppm of NH_3_. Reproduced from Wang, Z.; Yan, F.; Yu, Z.; Cao, H.; Ma, Z.; YeErKenTai, Z.N.; Li, Z.; Han, Y.; Zhu, Z. Fully Transient 3D Origami Paper-Based Ammonia Gas Sensor Obtained by Facile MXene Spray Coating. *ACS Sensors* **2024**, *9*, 1447–1457 [81].

Wang et al. came up with a fascinating way of developing 3D origami paper-based MXene NH_3_ gas sensors [81]. In this work, they used Ti_3_C_2_T*_x_* MXene/gelatin ink to create a high-performance, chemiresistive NH_3_ gas sensor intended for environmental and health-related biomarker monitoring. Using a basic spray-coating technique, the ink was utilized to print electrodes on a paper substrate. After stretching 1000 times, the resultant Ti_3_C_2_T*_x_*-based coating showed outstanding mechanical flexibility and adherence. With a response of 7% to 50 ppm NH_3_ and detectable concentrations ranging from 5 to 500 ppm, its porous structure improved NH_3_ sensing. Even after being stretched by 50%, the sensor kept its high performance and selectivity. Moreover, 3D mesostructured MXene origami that was made by mechanically guided compressive buckling showed promise for identifying the height distribution and direction of toxic gases. The origami paper made of MXene and gelatin is shown in Figure 7. These sensors are eco-friendly, entirely disintegrating in a PBS/H_2_O_2_/cellulase solution in 19 days, indicating that it is a good contender for wearable gas sensors that are high-performing, shape-morphable, and sustainable. Two-dimensional MXenes have become promising materials for gas sensors due to their abundant surface functional groups, excellent conductivity, and large surface area. Kong et al. explored 2D Ta_4_C_3_T*_x_* nanosheets for NH_3_ sensing [82]. Ta_4_C_3_T*_x_* nanosheets were synthesized using HF etching followed by TMAOH intercalation. The sensor was based on monolayer Ta_4_C_3_T*_x_* with abundant oxygen functionalities, which helps in exhibiting an exceptional response to NH_3_ gas at room temperature, surpassing other MXene-based sensors.

### 3.5. VOC Gas Sensors

A broad class of carbon-based compounds known as volatile organic compounds (VOCs) evaporate readily into the atmosphere. Paints, solvents, cleaning products, fuels, and construction materials are a few of the things that produce them, and they significantly raise both indoor and outdoor air pollution. VOCs can cause a variety of health problems, such as irritation of the eyes, nose, and throat as well as headaches, nausea, and more serious ailments such as damage to the liver, kidneys, and central nervous system. Furthermore, it is well recognized that some VOCs trigger carcinogenesis. Thus, VOC sensors play a crucial role in both monitoring and reducing exposure to these dangerous substances. Real-time measurement of VOC concentrations in interior spaces, workplaces, and industrial settings is made possible by VOC sensors.

Okechukwu et al. explored the use of a hybrid nanocomposite comprising tin oxide nanorods (SnO_2_-NRs) and GO for the chemiresistive detection of volatile compounds emitted by *Aspergillus flavus*, including hexanal, benzaldehyde, octanal, 1-octanol, and ethyl acetate vapors [30]. Three different kinds of sensors were developed: composites (SnO_2_-GO) and individual nanomaterials (SnO_2_ and GO). The study discovered that GO-based sensors were sensitive to several analyte vapors, although SnO_2_ NRs alone showed low sensitivity. The synergistic effect of integrating SnO_2_ NRs into GO layers improved sensor performance. The nanocomposite sensor showed superior sensitivity, selectivity, and moderate response and recovery times. Also, the performance sustained to 62% relative humidity (RH). These results highlighted the potential of SnO_2_ NRs-GO composite sensors for selective and sensitive detection of volatile chemicals, with substantial implications for environmental monitoring and food safety. He et al. used a microwave-assisted hydrothermal technique to create a highly sensitive and selective formaldehyde (H-CHO) gas sensor based on SnO_2_/Fe_2_O_3_ nanocomposites infused with rGO nanosheets [65]. With a rapid reaction time of 32 s and a recovery time of 33 s, the SnO_2_/Fe_2_O_3_@rGO sensor demonstrated a strong response of 11.21 to H-CHO gas as well as good reproducibility. This outperformed a SnO_2_@rGO sensor made of a single component. The heterojunction effect of SnO_2_, Fe_2_O_3_, and rGO, which promotes electron transport, is thought to be responsible for the increased gas response.

Kumar et al. developed a highly responsive and selective methanol gas sensor operating at room temperature using rGO/PANI nanocomposites [83]. The nanocomposites were synthesized by the modified Hummer’s method, a simple chemical oxidative synthesis process with camphor sulfonic acid. The sensors were tested for methanol vapor detection at concentrations of 50, 100, 150, and 200 ppm, and an 8 wt% rGO-doped polyaniline composite showed the highest response of 52% to 200 ppm. The sensors demonstrated good stability even after 180 days. Due to increasing environmental and safety concerns, advanced gas sensors for detecting volatile and hazardous organic substances are essential. Baker et al. conducted a study on the development of chemiresistive sensors using porous LIG to detect VOCs such as acetone and ethanol [19]. They produced flexible 3D porous LIG using femtosecond laser texturing of polyimide tape. By employing femtosecond pulsed laser deposition to decorate LIG with silver (Ag) and titanium oxide (TiO_x_) NPs, LIG-based heterojunction devices were developed to improve the sensor’s capabilities. The results demonstrated that decorating LIG sensors with Ag and TiO_x_ NPs enhanced their sensitivity, with Ag NPs exhibiting the best sensitivity. When exposed to VOC concentrations below 3000 ppm, the sensors showed a reversible response at room temperature. The enhanced availability of adsorption sites and the desirable work function of Ag are the key factors for the improved sensitivity of the Ag NPs-decorated porous LIG sensor. Shooshtari et al. developed vertically aligned CNT-based gas sensors to detect VOCs, with a focus on the impact of humidity on their sensing properties [84]. The investigation revealed that as the relative humidity increased from 10% to 80%, the electrical conductivity of the CNT sensors decreased by approximately 4%. However, when the humidity exceeded 80%, the conductivity began to increase slightly. Furthermore, it was found that humidity significantly affected the sensor’s response to VOCs, with a 40% reduction in sensitivity observed as the humidity increased up to 80%. This demonstrates the strong cross-sensitivity of CNT-based sensors to humidity, which poses a challenge for their use in real-world applications where humidity levels vary.

In the case of MXenes, Liu et al. utilized electrostatic adsorption and calcination strategies to create a Co_3_O_4_/MX7.5 heterojunction from ZIF-derived Co_3_O_4_ and alkalized Ti_3_C_2_T*_x_* MXene for ethanol gas sensing [85]. The Co_3_O_4_ NPs were found to uniformly grow on the surface of the alkalized Ti_3_C_2_T*_x_*, which has a lower work function and can transfer more electrons to Co_3_O_4_, enhancing the redox activity of Co^2+^. This increased activity helps adsorb more oxygen and produce more oxygen defects, enhancing the interaction between ethanol molecules and adsorption sites. The Co_3_O_4_/MX7.5 heterojunction-based sensors showed long-term stability for around 30 days, response of 3500%, and an LOD of 1 ppm. The sensors operated at a comparatively low temperature of 140 °C. Geng et al. used Cr_2_O_3_ nanoparticles derived from metal–organic frameworks (MOFs) to synthesize Cr_2_O_3_/Ti_3_C_2_T*_x_* MXene composites via hydrothermal and mechanical stirring methods [86]. Superior performance was demonstrated by the sensors based on Cr_2_O_3_/Ti_3_C_2_T*_x_* composites synthesized by the hydrothermal method. They exhibited a lower detection limit of 10.1 ppb, a shorter response/recovery time (90 s/210 s), better repeatability, and higher selectivity compared to the composites made by mechanical stirring. The sensors also showed a higher response of about 130.1 to 100 ppm n-butanol at a lower operating temperature of 160 °C. It was discovered that the majority of the MXene in the hydrothermally synthesized composites was oxidized to TiO_2_ and C-dots. The multiple heterojunctions in Cr_2_O_3_/Ti_3_C_2_T*_x_* composites and the C-dot electron sensitization effect added more electron transfer channels and active sites for gas diffusion and adsorption, greatly improving gas sensing performance. Karmakar et al. first demonstrated a flexible, room-temperature toluene gas sensor based on vanadium carbide (V_2_C) MXene [62]. The V_2_C MXene was produced by conventional HF etching. With a polyester substrate, the V_2_C-based sensor demonstrated strong cross-selectivity, long-term stability, rapid response time of 14 s and recovery time of 34 s, good repeatability, and an LOD of 47.85 ppb in the linear range of 5–200 ppm toluene at ambient temperature (27 ± 1 °C). In addition, the sensor showed a remarkable response of 775% to 200 ppm toluene and good selectivity against six additional hazardous gases that interfered. Its excellent electrical properties and abundance of surface functional groups (−F, −OH, and −O) are the reasons for its superior performance. The enhancement of toluene gas sensing performance through sulfur (S) doping on Ti_3_C_2_T*_x_* MXenes was reported by Shuvo et al. [87]. They explained the insufficient interlayer spacing between MXene nanoflakes can limit their effectiveness, but doping with heteroatoms like sulfur can overcome this challenge. When compared to their undoped counterparts, S-doped Ti_3_C_2_T*_x_* MXenes showed a significantly improved response, ranging from a 214% increase at 1 ppm to a 312% increase at 50 ppm. Additionally, the sensors demonstrated outstanding long-term stability and a noteworthy reactivity with 500 ppb toluene.

### 3.6. Humidity Sensors

Of numerous different sectors, humidity monitoring is important for plenty of reasons. Because high and low humidity may have detrimental effects on health, healthcare facilities must maintain ideal humidity levels to ensure patients’ comfort and avoid respiratory problems. Precise humidity management is critical in industrial environments to guarantee product durability and quality, especially in the food, pharmaceutical, and electronics sectors, where moisture can damage product integrity and manufacturing processes. Monitoring humidity is also essential to agriculture to maximize crop growth, inhibit mold growth, and maintain plant health. Furthermore, humidity control improves comfort, keeps mold from growing in houses, and keeps heating and cooling systems operating efficiently in everyday living spaces. Overall, accurate humidity monitoring is essential for health, safety, productivity, and comfort across diverse applications.

**Figure 8:**
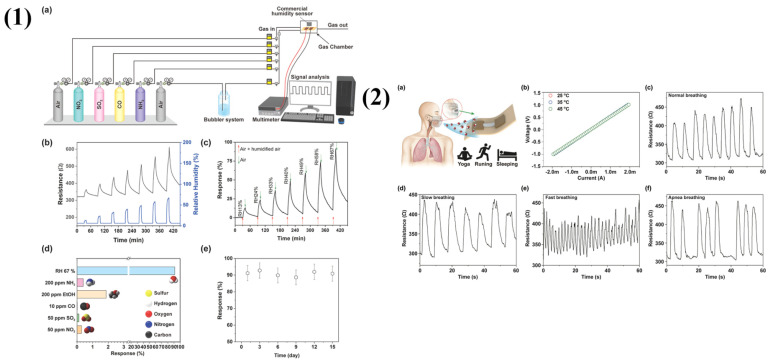
(**1**) Humidity sensing performance of LIG sensor. (**a**) Schematic representation of the experimental setup employed for gas sensing analysis. (**b**,**c**) Dynamic sensing response of the LIG sensor showcasing the variation in sensor resistance with respect to different RH levels. (**d**) Sensing response of the LIG sensor in the presence of other interfering gases such as NH_3_, EtOH, CO, SO_2_, and NO_2_. (**e**) Long-term stability of the LIG sensor (working temperature 25 °C, 67% RH). (**2**) (**a**) A schematic of breath sensing analysis. (**b**) Current versus voltage characteristics of the LIG sensor at three different temperatures. (**c**–**f**) Investigation of the LIG sensor’s response behavior under varying respiratory patterns, including slow, normal, fast, and alternating periods of respiration and apnea. Reproduced from Paeng, C.; Shanmugasundaram, A.; We, G.; Kim, T.; Park, J.; Lee, D.W.; Yim, C. Rapid and Flexible Humidity Sensor Based on Laser-Induced Graphene for Monitoring Human Respiration. *ACS Appl. Nano Mater.* **2024**, *7*, 4772–4783 [48].

Paeng et al. designed an LIG-based humidity sensor for respiratory monitoring and fabricated using a combination of laser irradiation and intense pulsed light (IPL) sintering techniques [48]. An ink comprising copper nanoparticles (CuNPs) and graphene nanoplatelets (GnPs) is coated to a polyimide (PI) substrate for fabricating the sensor. The PI layer is then exposed to laser irradiation to generate LIG. The use of conventional techniques like copper wire bonding or conductive paint application is no longer necessary since an IPL-sintered copper electrode provides reliable electrical contact. When the sensor’s relative humidity (RH) detection ability was tested in a range of RH values from 13% to 67%, the response increased from 15% to 92%. It showed low reaction to possible interfering gases such as ammonia, ethanol, carbon monoxide, sulfur dioxide, and nitrogen dioxide and a strong selectivity for RH (91.2%), as can be seen in Figure 8(1). Excellent repeatability was demonstrated as well across several cycles at 40% relative humidity. It consistently detected human breathing patterns for 30 min while monitoring a broad variety of respiratory patterns, including normal, slow, rapid, and apnea occurrences, as shown in Figure 8(2). These results demonstrate that LIG sensors can be integrated into contemporary medical procedures and used as cutting-edge instruments for clinical respiratory monitoring.

Yang et al. fabricated room-temperature planar-type gas and humidity sensors using Ti_3_C_2_T*_x_* MXene and alkalized Ti_3_C_2_T*_x_* through a dip-coating method [88]. Ti_3_C_2_T*_x_* MXene with an organ-like structure was synthesized from Ti_3_AlC_2_ (MAX phase) using the typical HF etching method, followed by alkaline treatment with NaOH to obtain alkalized Ti_3_C_2_T*_x_*. The intercalation of alkali metal ions (Na^+^) and the increased surface terminal oxygen-to-fluorine ratio ([O]/[F]) in Ti_3_C_2_T*_x_* significantly enhanced the humidity and gas-sensing properties at room temperature. The alkalized Ti_3_C_2_T*_x_* sensor demonstrated excellent humidity sensing capabilities, with approximately a 60-fold response signal change across an RH range of 11–95% and notable NH_3_ sensing performance (28.87% response to 100 ppm NH_3_). Table 2 below provides a comparison of sensor performance based on graphene and MXenes with respect to various gases.

### 3.7. Challenges and Solutions in the Fabrication of Sensors

Although there are some challenges in the process of fabricating heterostructure gas sensors on a large scale, there are also several promising alternatives. Integrating several materials with diverse qualities is a major problem that can lead to flaws or inadequate bonding. For precise control, this can be solved by employing sophisticated deposition processes such as chemical vapor deposition (CVD) and molecular beam epitaxy (MBE). Another problem is ensuring consistency over huge substrates, particularly when scaling up. Atomic layer deposition (ALD) and other automated deposition techniques with real-time monitoring aids help to preserve consistency and uniformity. Challenges involve the expense and complexity of the manufacturing procedures. However, they can be mitigated by utilizing scalable techniques like the sputtering process or simplifying the process by utilizing fewer functional layers. Although it is challenging to scale up nanostructures without sacrificing quality, self-assembly techniques and nanoimprint lithography (NIL) are promising alternatives. Nanostructures are often essential to sensor performance. Additionally, electrical contact between various layers in heterostructures can be troublesome. To cope with this, selective doping and other sophisticated contact engineering approaches might help. The employment of automated manufacturing systems, hybrid fabrication methods, alternative materials, and in situ monitoring for quality control will be essential for effectively scaling up the production. These obstacles may be overcome to manufacture heterostructure gas sensors at a larger scale with excellent dependability and performance. Improving long-term stability is a major problem since these sensors are vulnerable to environmental variables that might deteriorate their function over time, such as humidity, temperature variations, and contamination. For reliable, long-lasting operation, emerging technologies like machine learning-assisted gas detection and self-healing sensors may be necessary. The machine learning-assisted detection can improve the accuracy by identifying complex gas signatures, while self-healing sensors can regain functionality even after damage. Future gas-sensing applications utilizing graphene- and MXene-based materials may benefit from these developments by making them more resilient, scalable, and intelligent.

## 4. Gas Sensing Mechanism

Although MXene and graphene-based materials are widely used in gas sensing devices, they often exhibit distinct gas-sensing mechanisms due to their unique structural, chemical, and electronic properties. MXenes rely on surface adsorption and charge transfer interactions for gas sensing. They are distinguished by their two-dimensional structure, numerous surface functional groups, and high conductivity. A variety of surface terminations, such as -O, -OH, and -F, increase their reactivity with gas molecules and cause changes in electrical conductivity when the gas is adsorbed. Furthermore, it is possible to modify the interlayer spacing in MXenes to enhance sensitivity and gas diffusion. On the other hand, charge transfer processes between the gas molecules and the graphene surface are the primary means of gas detection for graphene-based materials, which are widely known for their large surface area, flexibility, and exceptional electrical capabilities. Increasing the number of active sites, defects, functional groups for gas interaction or heteroatom doping in graphene could further enhance its sensitivity and selectivity. The major difference is that graphene’s sensing performance is primarily determined by its pristine conductive network and the introduction of specific functional groups or defects, whereas MXenes greatly benefit from their tunable surface chemistries and structural modifications, which can be specifically designed for target gas interactions.

**Figure 9:**
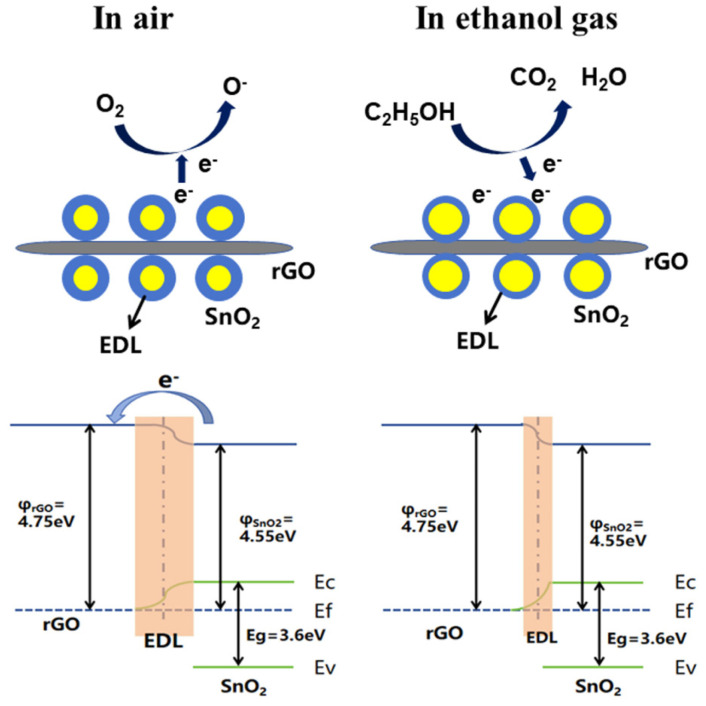
Schematic illustration of the gas sensing process of rGO/SnO_2_ nanocomposites. Reproduced from An, D.; Dai, J.; Zhang, Z.; Wang, Y.; Liu, N.; Zou, Y. rGO/SnO_2_ Nanocomposite Based Sensor for Ethanol Detection under Low Temperature. *Ceram. Int.* **2024**, *50*, 16272–16283 [59].

Motora et al. explained the gas sensing mechanism of metal oxide-based sensors, particularly for H_2_ gas, which relies on changes in electron depletion layers (EDLs) and oxygen adsorption [92]. For the NiSe_2_-rGO composite sensor, the mechanism involves oxygen adsorption of NiSe_2_ from the surrounding air, which extracts electrons from its conduction band, increasing the EDL. Sensing is vitalized and the electrical conductivity increases when NiSe_2_ is exposed to H_2_ gas because hydrogen gas interacts with the adsorbed oxygen species and releases electrons back into the conduction band. Due to its high electron mobility, reduced graphene oxide (rGO) in the composite enhances the sensing capability by functioning as an electron acceptor and aiding charge carrier movement. The enhanced performance of the sensor is mostly due to the synergistic interaction between rGO and NiSe_2_. An et al. discussed the gas sensing mechanism of SnO_2_-based sensors towards ethanol [59]. The gas sensing originates from resistance changes caused by gas adsorption and reactions on the material’s surface. In the presence of air, oxygen captures electrons from SnO_2_, forming adsorbed oxygen ions (O_2_^−^, O^−^, and O^2−^) that increase resistance. Ethanol molecules, which are a reducing agent, provide electrons to SnO_2_, hence lowering resistance and shrinking the depletion layer. O^2−^ and O^−^ are the primary oxygen ions that are seen mostly at 120 °C. SnO_2_ nanoparticles adhere to rGO nanosheets in rGO/SnO_2_ composites, improving gas adsorption and raising active sites, although too much rGO can counteract this effect. At the p-n heterojunction, a 0.2 eV band bending is produced by the distinct work functions of SnO_2_ and rGO, as shown in Figure 9, which modulates the electron density and enhances gas responsiveness. Since rGO/SnO_2_ sensors have a 20-fold resistance increase because of their smaller particle size, larger surface area, more flaws, and possible barriers that might facilitate gas reactions, they exhibit much-improved ethanol detection at lower temperatures when compared to pure SnO_2_ sensors.

In the case of MXenes, Gasso et al. developed a WO_3_/Ti_3_C_2_T*_x_* (WM) nanocomposite that outperforms pure WO_3_ NRs and Ti_3_C_2_T*_x_* in gas sensing performance due to the synergistic effects of WO_3_ and Ti_3_C_2_T*_x_* [93]. The high surface area of the WM sensor and the highly conductive Ti_3_C_2_T*_x_* platform enhance gas molecule adsorption and facilitate charge carrier transport, thereby improving gas sensing performance. The heterojunction between WO_3_ (*φ* = 4.8 eV) and Ti_3_C_2_T*_x_* (*φ* = 3.9 eV) creates a Schottky barrier with n-type response behavior, generating electron depletion and hole accumulation layers (HAL) at the interfaces. Atmospheric oxygen adsorbs at these layers, trapping electrons and widening the EDL. When exposed to NO_2_ gas, the oxidizing NO_2_ captures electrons from the WO_3_ conduction band, further widening the EDL and increasing sensor resistance. This process involves both monomolecular and dissociative adsorption mechanisms, where NO_2_ either directly captures electrons or interacts with oxygen-deficient centers to form adsorbed oxygen species. Flushing out NO_2_ and exposing the sensor to dry air release electrons, decreasing resistance. The WO_3_ NR’s high surface area and Ti_3_C_2_T*_x_*’s good conductivity, along with the formation of heterojunctions, enable rapid response and recovery, which are favorable for room-temperature sensing. Additionally, sodium L-ascorbate (SA) treatment on Ti_3_C_2_T*_x_* in the SA-WM nanocomposite sensor mitigates humidity effects, enhancing adaptability to real-time sensing applications and prolonging shelf life. Chen et al. demonstrated a facile, robust route of functionalizing Ti_3_C_2_T_x_ MXenes with a superhydrophobic protection layer by employing fluoroalkyl silane (FOTS) functionalization [60]. The end-functional groups can regulate the surface characteristics, according to a systematic study of Ti_3_C_2_T*_x_* surfaces treated with self-assembled monolayers (SAM). FOTS-functionalized Ti_3_C_2_T*_x_* exhibits high tolerance in strongly acidic and basic solutions in addition to hydration stability in humid settings. The sensing mechanisms for MXene-based sensors could be understood by comparing the sensing behavior of the Ti_3_C_2_T*_x_*-F-based sensor toward strong oxidizing (electron-accepting) NO_2_ and electron-donating VOCs (acetone, ethanol, etc.). This sensor showed comprehensive positive variations of resistance regardless of gas type, indicating that its charge-carrier transport channel is always hindered when a gas molecule is adsorbed. Such behavior is distinctive from other 2D semiconducting materials, where the resistance change depends on the electron-donating or accepting properties of gas molecules and the type (p- or n-type) of channel materials. The comprehensive positive response of the MXene-based sensor is due to the metallic conductivity of Ti_3_C_2_T*_x_*, where gas adsorption reduces the mobility of charge carriers, thereby increasing the channel resistance. The enhancement in gas sensing performance of Ti_3_C_2_T*_x_*-F most likely originates from its significantly increased interlayer distance and surface area compared to pristine Ti_3_C_2_T*_x_* and Ti_3_C_2_T*_x_*-Cl. This study shows how the surface functionality works in gas detection and improves material stability.

**Figure 10:**
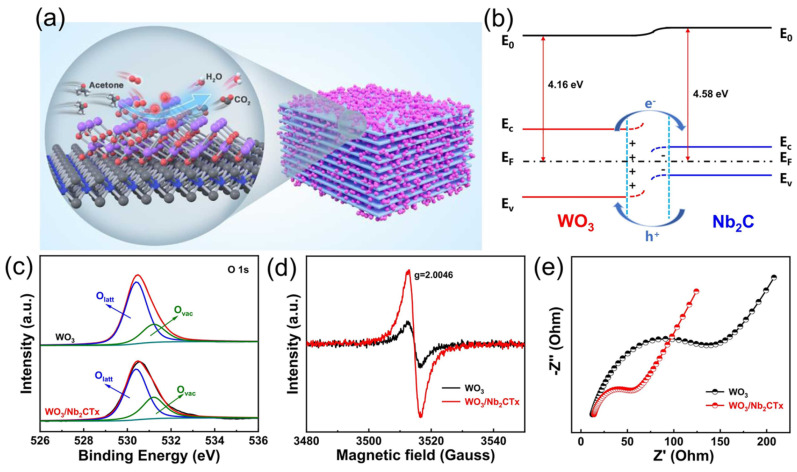
(**a**) Schematic illustration of the sensing mechanism for the WO_3_/Nb_2_CT*_x_* heterojunction sensor. (**b**) The energy band configuration of WO_3_/Nb_2_CT*_x_* heterojunction (E_F_ denotes the Fermi level, and E_C_ and E_V_ are the conduction band edge and valence band edge, respectively). (**c**) XPS spectra of O 1s for WO_3_ and WO_3_/Nb_2_CT*_x_* heterojunction. (**d**) EPR spectra of WO_3_ and WO_3_/Nb_2_CT*_x_* heterojunction. (**e**) EIS spectra of WO_3_ and WO_3_/Nb_2_CT*_x_* heterojunction. Reproduced from Wang, P.; Guo, S.; Zhao, Y.; Hu, Z.; Tang, Y.; Zhou, L.; Li, T.; Li, H.Y.; Liu, H. WO_3_ Nanoparticles Supported by Nb_2_CT*_x_* MXene for Superior Acetone Detection under High Humidity. *Sensors Actuators B Chem.* **2024**, *398*, 134710 [64].

A detailed sensing mechanism of WO_3_/Nb_2_CT*_x_* towards acetone gas was provided by Wang et al. [64]. The ability of gas adsorption and sensing is improved by oxygen vacancies on the surface of sensing materials. They proved the existence of these vacancies in WO_3_/Nb_2_CT*_x_* using electron paramagnetic resonance (EPR) spectroscopy and X-ray photoelectron spectroscopy (XPS). The sensors made of WO_3_ and WO_3_/Nb_2_CT*_x_* both exhibit characteristics of an n-type semiconductor. Negative oxygen species are generated in the atmosphere because conduction band electrons are captured by adsorbed oxygen molecules. When exposed to acetone, these oxygen species react with acetone molecules, releasing electrons back into the conduction band and reducing sensor resistance. The heterojunction structure of WO_3_/Nb_2_CT*_x_* prevents WO_3_ nanoparticle aggregation and exposes more active sites, increasing the surface area for gas adsorption. Nb_2_CT*_x_* MXene’s framework significantly improves acetone sensitivity by increasing surface area and oxygen vacancy ratio. The band bending induced at the WO_3_/Nb_2_CT*_x_* heterojunction facilitates charge transfer and improves gas sensing capability. The sensitivity and detection limits of gas sensors are greatly impacted by band bending at heterojunctions, which modifies the electrical characteristics at the interface between different materials. Upon contact between materials with different band gaps or work functions, the energy bands of the materials are bent to align at the Fermi level, forming an accumulation or depletion region. This band bending modifies the nature of charge-carrier transport, and interactions between gas molecules and the sensor surface modulate the potential barrier height, resulting in noticeable variations in conductance or resistance that raise sensitivity. Furthermore, the distinct depletion region at the heterojunction enhances the signal-to-noise ratio, enabling the detection of even minute quantities of gases and therefore reducing the detection limits. Electrochemical impedance spectroscopy (EIS) validates this heterojunction, demonstrating enhanced charge transport because of interfacial electric field, which is illustrated in Figure 10. As the combined effects of these elements, the WO_3_/Nb_2_CT*_x_* sensor shows better acetone gas detection capability.

## 5. Conclusions

This review presents a comparative analysis of the synthesis and sensor fabrication procedures of graphene and MXene for gas sensing applications. Flexible, rigid prototypes that detect gases at varying concentrations have been developed by integrating various physical forms of graphene and MXene structures with polymers, metal oxides, and other semiconducting materials. These sensors have been extensively evaluated in various environments. The advantages of MXene-based sensors are their great selectivity and sensitivity because of their surface functionalities. Since graphene lacks these surface functional groups, other materials are required to alter the surface characteristics of graphene to enhance an interaction with gas molecules. Graphene has extremely high conductivity and network due to its unique structure, which results in a high surface area for interaction and faster response by cutting the operating time. Combining these sensor technologies with wireless connectivity would also make it easier for the prototypes to be used at predetermined intervals in industrial and residential settings. The remarkable properties of graphene and MXene-based gas sensors can make the environment safer and raise the standard of living for people. Even though they have lots of advantages, they also have certain limits. Regarding real-time applications, partial recovery is a typical drawback for graphene-based gas sensors. In brief, molecules that are physically adsorbed tend to adhere to surfaces, occupying adsorption sites, thereby reducing the available sites for incoming molecules. The device’s sensitivity decreases with repeating exposure cycle to the analyte due to the incomplete recovery. In addition, oxidation can cause MXenes to lose their high electrical conductivity, making them less effective in real-time applications for gas sensing. This is especially true when the material is exposed to oxygen and humidity. Dedicated research is ongoing to tackle these issues. Future research may focus on creating multifunctional sensors capable of detecting multiple gases, scaling up material synthesis process for cost-effective mass production, integrating advanced data analytics and machine learning for acute data interpretation, and assessing the environmental and health impacts of the sensing materials. Furthermore, investigating the synergies between cutting-edge technologies like flexible electronics, self-healing technology, and nanotechnology will spur additional innovation and lead to more flexible and reliable gas sensing systems.

## Figures and Tables

**Table 1:** Comparison of synthesis methods of graphene- and MXene-based composites and their advantages and disadvantages.

Target Gas	Sensing Materials	Synthesis Methods	Advantages	Disadvantages	Response/Recovery Time (s)	Ref.
CO_2_	Graphene OP	Oxygen plasma treatment	Effective surface cleaning	Equipment costs	300/900	[3]
	Ti_3_C_2_T_x_/PANI-PP	HF etching and in situ polymerization	Controlled etch rate and reduced processing steps	Toxic and process complexity	115/26	[36]
NO_2_	rGO/α-Fe_2_O_3_	Solvothermal method	Low temperature	Long reaction times	32.42/-	[29]
	2D-0D MXene/PbS	Etching and in situ formation	Precise and direct formation	Stringent handling and difficult to precisely control	27/91	[35]
H_2_S	Graphene aerogels	Hydrothermal reduction and chemical reduction	Controlled morphology and simple	Difficult to monitor and unwanted by-products	12 and 13/134 and 165	[10]
	3D Porous MXene Nanosheet/ SnO_2_	HCl-LiF etching and hydrothermal	High selectivity and versatility	Difficult to control and high energy consumption	44.2/266.7	[9]
NH_3_	PVC/rGO	Improved hummers method	Higher yield	Over-oxidation	46/88	[37]
	Ti_3_C_2_T_*x*_/TiO_2_	Selective etching	Highly precise	Surface damage	205/110	[58]
C_2_H_5_OH	rGO/SnO_2_	Hydrothermal method	Formation of metastable phases	High-pressure equipment	-	[59]
	Ti_3_C_2_T*_x_*-F	HCl-LiF etching	High selectivity	Slow reaction times	39/139	[60]
Toluene	3D TiO_2_/G-CNT)	CVD and sparking method	High purity and rapid processing	High equipment costs and particle aggregation	9/11	[61]
	V_2_C MXene	HF etching	High precision	Hazardous	14/34	[62]
Acetone	SnO_2_–RGO	Hydrothermal treatment	Eco-friendly process	Limited solvent options	107/95	[63]
	WO_3_/Nb_2_CT*_x_*	HF etching and hydrolysis calcination	Versatility and simple	Safety risks and limited control over particle size	9/881	[64]
HCHO	SnO_2_/Fe_2_O_3_-rGO	Microwave-assisted hydrothermal method	Very fast and uniform heating	Challenges with scale up	32/33	[65]
	MXene/Co_3_O_4_	Etching and hydrothermal	Functionality and controlled morphology	Safety concerns and limited scalability	83/5	[66]
H_2_	PdNP-decorated 3D graphene	MOCVD	High-quality thin films	Material waste	8/38	[67]
	MXene-Pd CNC film	HCL-LiF etching and colloidal solution-based vacuum filtration	Room-temperature processing and cost-effective	Corrosive chemicals and clogging	32/161	[68]

**Table 2:** Performance comparison of graphene- and MXene-based gas sensors to various gases.

Target Gas	Sensing Materials	Sensitivity (Maximum)	Operating Range (ppm)	Response Time (s)	Recovery Time (s)	Operating Temp. (°C)	Ref.
CO_2_	Perovskite—graphene	0.9	20–100	300	900	RT	[16]
	Ti_3_C_2_T_x_/PANI-PP	0.0304%/ppm	25–1500	115	26	RT	[45]
NO_2_	CuO/rGO	200.8%/ppm	1–5	9	110	RT	[29]
	CuO NPs/Ti_3_C_2_T_x_	~46	0.03–100	2.84	33.5	RT	[89]
H_2_S	Graphene HR and CR aerogels	0.255 and 0.3984/ppm	1–20	43.25 and 40.5	329.75 and 519	RT	[2]
	Ti_3_C_2_T_x_/Zn_2_SnO_4_-5	110	0.01–8	14	230	RT	[27]
NH_3_	PANI/PNGN 15%	8.1%/ppm	0.25–100	21	56	RT	[13]
	MNPA-100	1.85%/ppm	10–100	231.2	165.4	RT	[26]
C_2_H_5_OH	3.3% rGO/SnO_2_	48.3	1–200	-	-	120	[28]
	2H-MoS_2_/Ti_3_C_2_T_x_ MXene	0.4%/ppm	50–1000	5	12	140	[1]
Toluene	3D TiO_2_/G-CNT	42%	50–500	9	11	RT	[61]
	S-Ti_3_C_2_T*_x_*	59.1%	1–50	-	-	RT	[87]
Acetone	SnO_2_–RGO	-	10–2000	107	95	RT	[63]
	Ti_3_C_2_T*_x_*/SnO-SnO_2_	12.1	10–100	18	9	RT	[90]
HCHO	MoS_2_/graphene	61%	0–500	22	35	RT	[91]
	Ti_3_C_2_T*_x_*/Co_3_O_4_	9.2%	0.01–10	83	5	RT	[66]
H_2_	PdNP-decorated 3D graphene	41.9%	0.1–3%/air	8	38	30–150	[67]
	Ti_3_C_2_T*_x_*/Pd	23%	0.5–40%/air	32	161	RT	[68]

## Data Availability

The data presented in this study are available on request from the corresponding author.
